# Exploring the impact of emergency risk information on construction workers’ emergency information behavior: insights from confirmatory and exploratory PLS-SEM analyses

**DOI:** 10.3389/fpubh.2025.1670767

**Published:** 2025-09-24

**Authors:** Hui Liu, Rui Zhang, Xiaodi Gao, Ran Jing, Zhichao Zhang, Xinyue Miao

**Affiliations:** ^1^School of Emergency Science and Engineering, Jilin Jianzhu University, Changchun, China; ^2^School of Urban Construction, Changchun University of Architecture and Civil Engineering, Changchun, China

**Keywords:** emergencies, risk information, emergency information behavior, construction workers, PLS-SEM

## Abstract

Drawing on risk perception and information adoption theories, this study develops a structural equation model to examine the factors influencing emergency information behavior (EIB). The model includes risk information characteristics (RIC) and information dissemination channels (IDC) as independent variables, risk perception (RP) and information self-efficacy (ISE) as mediating variables, and EIB as the dependent variable. Data were collected from a questionnaire survey of 569 construction workers in Hangzhou and analyzed using Partial Least Squares SEM (PLS-SEM). The results indicate that RIC and IDC have a significant positive influence on RP, ISE, and EIB. Risk perception directly promotes EIB and indirectly influences it by enhancing information self-efficacy. Information self-efficacy, in turn, is also a significant driver of EIB, with the complete model explaining 55.5% of its variance (*R*^2^ = 0.555). This study concludes that delivering high-quality risk information through multiple channels effectively enhances workers’ ability to retrieve and use information during emergencies. This is achieved by improving their risk perception and information self-efficacy. The findings provide valuable empirical evidence and strategic recommendations for emergency management in the construction industry.

## 1 Introduction

In recent years, the construction industry has continued to develop. With the acceleration of urbanization and the expansion of the construction industry, safety risks at construction sites have significantly increased. The construction industry is a crucial pillar of the national economy, but its characteristics—such as open-air operations, high-intensity labor, and complex environments—expose construction workers to high-risk emergencies (e.g., site collapses, machinery accidents, extreme weather disasters) (1). These risks are predominantly dynamic in nature, characterized by sudden onset, rapid escalation, and context-dependent evolution (e.g., structural instability triggered by weather changes, unanticipated mechanical failures). Unlike static risks (e.g., chronic exposure to noise), dynamic risks require real-time information adaptation to mitigate immediate threats (2). Emergencies refer to sudden natural disasters, accident disasters, public health events, and social safety incidents (3), characterized by suddenness, uncertainty, catastrophic consequences, and negative impacts (4), which can significantly affect social order and stability. According to the International Labour Organization (ILO), approximately 20% of global occupational fatalities occur in construction, with over 60% directly related to delayed emergency responses in emergencies (ILO, 2023). Simultaneously, construction workers generally face limitations in information access: lower education levels, high mobility, and reliance on oral information transmission often leave them in “information islands” during emergencies, exacerbating personal safety and mental health risks.

With the promotion of smart construction site technologies, risk information dissemination methods (e.g., IoT warnings, mobile push notifications) are evolving (5). However, existing research primarily focuses on emergency behavior patterns of the general public, neglecting the unique characteristics of high-risk occupational groups like construction workers. For instance, issues such as difficulty receiving audio alerts in noisy environments or processing complex information during high-intensity work have not been incorporated into mainstream risk communication theoretical frameworks.

Research on emergency information behavior in emergency contexts has formed a multi-dimensional theoretical framework, but contextualized studies targeting specific high-risk occupational groups remain insufficient. Ahmed et al. (6) based on the global public health crisis of COVID-19, revealed the interaction mechanism between emotional states and risk perception on public information behavior, confirming that negative emotions significantly amplify cognitive biases in risk perception, thereby altering information acquisition strategies. Chisty et al. (7) demonstrated that risk perception during emergencies can be amplified or mitigated through information-seeking activities, indicating a dynamic relationship between perceived threat levels and information behavior. This study emphasized that information search can both raise awareness and potentially reduce perceived risk depending on the nature of the information obtained. At the social media dissemination level, You et al. (8) found that during the early stages of COVID-19 in South Korea, emergency alert text messages effectively promoted preventive measures, highlighting the importance of timely and accessible information dissemination during emergencies. Li et al. (9) and Ayala et al. (10) investigate cognitive and perceptual processes under emergency or complex conditions, such as autonomous driving and aviation tasks. Their findings demonstrate that under emergency or high-stakes situations, rapid information processing and integration are vital, with brain region activation and gaze behavior serving as indicators of situational awareness and cognitive load. These insights are relevant for understanding how emergency responders and individuals process critical information during crises. Carr et al. (11) further explored the incentive effects of risk information on protective behavior. Their research showed that in coastal storm warnings, timely, clear, and instructive communication methods (e.g., scenario simulation briefings) significantly improved residents’ disaster preparedness levels, underscoring the strategic value of communication media and time. Xu et al. (12) used data mining techniques to analyze public attribute preferences in emergency decision-making, with their group clustering method achieving precise targeting of risk information. Mao et al. (13) proposed a decision-making framework based on cumulative prospect theory to select appropriate epidemic prevention strategies. Their approach considered psychological factors such as risk preferences and loss aversion, indicating that effective risk information must account for human decision biases to optimize emergency responses. Wang et al. (14) studied social media communication behaviors of Chinese mobile users during the pandemic, noting that cultural context and censorship influenced information seeking and sharing, thereby affecting collective situational awareness and social resilience. Focusing on individual information behavior characteristics, Xie et al. (15) examined the role of social media as a communication channel in public emergencies, finding that Chinese social media users actively participated in discussions, sharing, and expressing opinions during crises. This participatory communication behavior facilitated information dissemination and collective awareness, an essential component of emergency information behavior. Zhang et al. (16) studied how path turning angles in virtual environments affect individuals’ perception of emergency signs and their compliance, suggesting that spatial and perceptual factors influence information interpretation and subsequent actions during evacuation. Vinnell et al. (17) extended this understanding to natural disasters, exploring residents’ evacuation decisions after earthquakes and emphasizing that immediate information needs and motivations are crucial in shaping evacuation behavior. Huff et al. (18) showed that emotionally evocative patient behaviors could influence emergency nurses’ assessments and handovers, indicating that emotional cues are significant in clinical decision-making during emergencies. Additionally, Lappin et al. (19) explored the mental health aspects of emergency information behavior, investigating how digital mental health services could be introduced to individuals with self-harm or suicidal behaviors, suggesting that accessible information and intervention options are vital for managing mental health crises during emergencies.

While existing research has established a foundational “environment-psychology-behavior” analytical paradigm, a critical synthesis reveals two significant limitations that impede its direct application to high-risk industries like construction. First, the majority of case studies, such as those by Ahmed et al. (6) on COVID-19 and Vinnell et al. (17) on earthquakes, focus on large-scale public health events and natural disasters. While these studies provide valuable insights, their findings are not readily transferable to the localized, dynamic, and often sudden emergencies characteristic of construction sites (e.g., site collapses, machinery accidents), which evolve under entirely different temporal and spatial constraints. Second, existing models are predominantly tailored to the general public, thereby failing to account for the unique occupational ecosystem of construction workers. This group operates in environments characterized by high noise levels, significant labor mobility, and a strong reliance on oral communication channels, often leaving them in “information islands” during emergencies. Generic risk communication frameworks frequently overlook how these contextual factors exacerbate personal safety risks by impeding the effective reception and processing of critical information.

This study aims to address this “cognition-environment-behavior” imbalance by developing and validating a model tailored specifically to construction workers. Our model provides a more nuanced understanding than generic frameworks. It fills a critical academic gap while also providing actionable solutions for policymakers, construction companies, and workers.

## 2 Theoretical foundations

### 2.1 Risk perception theory

“Risk” manifests in two primary forms: a realized, quantifiable technical definition measured by indicators such as casualty counts and economic losses, and risk perception—a psychological state between reality and imagination—whose importance in contemporary society is increasingly prominent (20). With rapid economic development and escalating social and natural conflicts, global emergencies occur frequently, exhibiting trends of deep harm and wide scope (21). In the new era, public awareness of emergency management and demand for emergency information have reached unprecedented heights.

The concept of risk perception originated in psychology. As technology evolved and environments became more complex, risk perception gradually extended to communication, information resource management, and risk management fields (22). Current scholars analyze risk perception from definitions, dimensional divisions (23–25), and impact levels. Regarding definitions, scholars generally agree that risk perception is a psychological cognition of potential risks and an estimation/judgment of possible negative impacts (e.g., illness, injury) influenced by education level, emotional changes, socio-cultural factors (26, 27). In dimensional division, early scholars focused on the formation and impact of financial, social, functional, physical, and psychological risk perceptions (28–30). With the continuous development of information technology, risk perception has gained increasing attention (31). At the impact level, cultural literacy, information quality, online contexts, and website usage experiences can induce different dimensions of risk perception (32–35), leading to changes in information sharing (36), information seeking (37, 38), risk response (39), and continuance usage (40) behaviors and intentions.

Risk perception plays a key role in emergency information behavior among construction workers (7). First, it strengthens workers’ information awareness and response capabilities. When construction workers accurately perceive risks at construction sites, they proactively attend to safety warnings, correctly assess threat levels from accidents like collapses and falls, thereby enhancing their ability to identify safety signals and comply with operational norms (41). Second, it promotes information coordination efficiency on-site. Clear risk cognition drives workers to promptly transmit hazard information through team communication networks while strictly following information reporting procedures in emergency plans, establishing a site-level emergency information response mechanism. This perception mechanism also enhances workers’ ability to interpret professional safety instructions, enabling them to accurately capture critical safety broadcasts in noisy, multi-process environments and avoid secondary accidents caused by information misjudgment (42).

### 2.2 Information adoption theory

Information Adoption Theory (IAT) was first proposed by Sussman and Siegal (43). Its core idea is that the process by which information influences decision-making can be viewed as an information adoption process, with information usefulness as the antecedent variable determined jointly by information attributes and recipient behaviors. As an important theory influencing user decisions in online information dissemination contexts, IAT has been widely applied in studies on factors affecting information usefulness, information adoption, and purchasing decisions.

**Figure 1:**
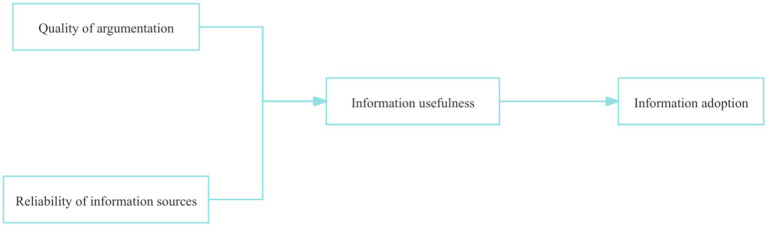
Information adoption model.

To explain how people evaluate and adopt advice or information, Davis (44) drew on the perceived usefulness variable from the Technology Acceptance Model, proposing information usefulness as the antecedent variable influencing information adoption. Simultaneously, incorporating ideas from the Elaboration Likelihood Model (45)—central route (argument quality) and peripheral route (information source credibility)—they established the initial Information Adoption Model (IAM) comprising argument quality, source credibility, information usefulness, and information adoption, as shown in Figure 1. The model indicates that argument quality and source credibility positively influence information usefulness, which in turn positively affects information adoption. Argument quality refers to the persuasiveness of arguments in information (46)^,^ reflecting recipients’ perception of information content itself, measured by information completeness, consistency, and accuracy. Source credibility is the degree to which recipients perceive the source as effective, credible, and trustworthy (47), measured by the author’s expertise, trustworthiness, and reliability. Information usefulness is the perceived utility of information (48), a dependent variable of adoption behavior. Information adoption is the process where recipients evaluate information, find it meaningful, and adopt its content (49), representing the internalization stage of knowledge transfer where explicit information transforms into internal knowledge and meaning (50).

## 3 Theoretical model construction and research hypotheses

### 3.1 Independent variables

This study’s independent variables are risk information characteristics (RIC) and information dissemination channels (IDC).

Risk information characteristics refer to key attributes or features of information used to describe, assess, and manage risks (51). These characteristics ensure risk information is effective, reliable, and practical in decision-making (52). Based on a cross-perspective of risk management theory (53) and information science (54), this study systematically divides RIC into (55) four core dimensions: information accuracy (objective truthfulness reflecting risk essence), information timeliness (temporal responsiveness capturing risk dynamics), information completeness (comprehensive coverage of risk elements), and information credibility (source reliability and traceability).

Information dissemination channels are paths, media, or platforms through which information flows from senders to recipients (56). In risk management and modern information ecosystems, channel selection directly affects information accessibility, credibility, and dissemination efficiency, serving as a critical hub connecting information production and application. Based on source authority, dissemination mechanisms, and audience trust levels (57), channels are categorized into four types: official media, social media, interpersonal communication, and professional institutions. This classification reflects not only media properties but also the social construction process of risk cognition (58).

### 3.2 Mediating variables

This study’s mediating variables are risk perception (RP) and information self-efficacy (ISE).

Risk perception is the subjective cognition and judgment of potential dangers or uncertainties by individuals or groups (59). It reflects how individuals understand, assess, and respond to risks—judgments often influenced by psychological, social, cultural, and experiential factors. Unlike objective risk assessment, RP emphasizes subjective feelings, leading to significant variations in risk judgments (60). When individuals/groups receive threatening risk information, they assess perceived threat levels (61). Thus, RP mediates the impact of risk information on workers’ emergency information behavior.

As a multi-dimensional psychological cognitive process, the operationalization of Risk Perception (RP) in this study is grounded in established frameworks. Its four core dimensions are adapted from the foundational cognitive-affective model by Trumbo et al. (62) and tailored to the construction safety context. These dimensions are threat severity perception, which is the objective assessment of potential damage; personal vulnerability perception, a rational analysis of susceptibility to harm; event urgency perception, the temporal awareness of risk evolution; and controllability perception, the subjective belief in one’s ability to respond.

Information self-efficacy refers to an individual’s or group’s assessment of their ability to implement countermeasures when perceiving threats (63), a key factor influencing proactive information processing. As a core competency for processing information, the four-module structure of ISE is derived from established literature on the topic. The dimensions are directly informed by the conceptual work of Serap Kurbanoglu (64) and the empirical research of Yan et al. (65). They consist of information comprehension, which involves effective identification and interpretation; information discrimination, entailing authenticity verification and filtering; information application, or the transformation of knowledge; and information adaptation, which relates to responsiveness and resilience.

### 3.3 Dependent variables

This study’s dependent variable is emergency information behavior (EIB).

The EIB refers to the process of individuals interacting with emergency information resources (66). Based on literature and risk information features, this study defines EIB according to two principles: information content/form must be actionable by workers, and behavior must be relevant to workers (67). It divides EIB into four dimensions: emergency information seeking, sharing, verification, and compliance (68).

Emergency information seeking involves activities to find, collect, and request emergency information, influenced by workers’ information literacy, abilities, psychological traits, and information environments (7). Emergency information sharing features storing, comparing, selecting, and processing information to form utilizable resources (69). Emergency information verification entails authenticating conflicting/ambiguous information (e.g., contradictory instructions, unverified warnings) through cross-checking official notices, consulting safety officers, or on-site hazard observation (70). Emergency information compliance involves executing protective actions based on credible information—evacuating per instructions, wearing emergency gear, complying with work stoppages—directly impacting life safety (71).

### 3.4 Model construction and research hypotheses

Our theoretical framework is designed to systematically examine the full spectrum of relationships through which emergency information influences construction workers’ behavior. To achieve this, we conceptualize and test three distinct types of effects, each represented by a specific set of hypotheses:

1. Indirect Effects via Cognitive Mediation: The core of our model posits that informational factors (RIC and IDC) shape behavior (EIB) indirectly by influencing cognitive mediators (RP and ISE). Hypotheses H1 through H7 are formulated to test this central mediational chain.
2. Direct Effects on Behavior: Beyond the cognitive pathway, we also hypothesize that informational factors may have a direct, independent impact on behavior. For instance, clear and authoritative information might trigger immediate compliance without deep cognitive processing. Hypotheses H8 and H9 are specifically designed to test for the existence and magnitude of these direct effects.
3. Antecedent Relationship Among Exogenous Variables: Finally, we examine the relationship between our two primary predictors. In risk communication, the quality of information content (RIC) is a critical antecedent to its dissemination through effective channels (IDC). Hypothesis H10 is included to test this foundational relationship within the information ecosystem.

By testing these three sets of hypotheses, our study can provide a comprehensive and decomposed understanding of the overall influence mechanism. However, the relationship between risk perception and information self-efficacy warrants special consideration, as competing theoretical perspectives exist. On one hand, a heightened sense of risk can motivate individuals to seek and process information more proactively, thereby enhancing their confidence and self-efficacy. On the other hand, literature in cognitive psychology and crisis communication suggests that excessively high levels of perceived risk can induce anxiety and cognitive overload (72). This emotional distress may impair an individual’s judgment and diminish their belief in their ability to effectively manage complex information. In the high-pressure context of a construction site emergency, it is plausible that the overwhelming nature of a threat could negatively impact a worker’s perceived information efficacy. Therefore, the direction of this relationship is not unequivocally positive, making it a critical dynamic to test empirically. Accordingly, the following research hypotheses are proposed:

*H1*: Risk information characteristics have a positive effect on risk perception.

*H2*: Risk information characteristics have a positive effect on perceptions of information efficacy.

*H3*: Information dissemination channels have a positive effect on risk perception.

*H4*: Information dissemination channels have a positive effect on perceived information efficacy.

*H5*: Risk perception has a positive effect on emergency information behavior.

*H6*: Sense of information efficacy has a positive effect on emergency information behavior.

*H7*: Perceived risk has a negative effect on perceived information efficacy.

*H8*: There is a direct positive effect of risk information characteristics on emergency information behavior.

*H9*: There is a direct positive effect of information dissemination channel on emergency information behavior.

*H10*: There is a positive effect of risk information characteristics on risk communication channels.

This study proposes a theoretical framework, grounded in Risk Perception Theory and Information Adoption Theory, to investigate how emergency risk information shapes construction workers’ emergency information behavior.

**Figure 2:**
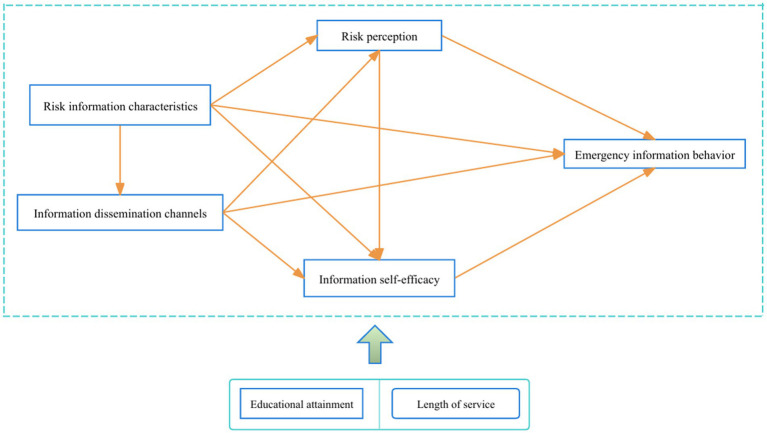
Theoretical structural equation model.

The structural equation modeling was constructed as shown in Figure 2.

## 4 Materials and methods

### 4.1 Research design

This study used quantitative analysis to test the hypothesis model in Figure 2. Questionnaire-based quantitative analysis is efficient with sufficient time and low budgets (73). A cross-sectional data collection method was adopted as it achieves research objectives more easily, requires no personal identification, and allows free responses (74). Stratified random sampling ensured sample diversity and representativeness (75). Workers were stratified by construction project participation, with random samples drawn from each stratum. This strategy captured responses from diverse backgrounds, enhancing generalizability.

The first part of the questionnaire measured the demographic characteristics of the respondents, including gender, age, length of service, education level, and type of work. The second part was the main part of the questionnaire, which consisted of the Risk Information Characteristics Scale, Information Dissemination Channels Scale, Risk Perception Scale, Information Efficacy Scale, and Emergency Information Behavior Scale, and a five-point Likert scale was used to measure all the items, where “1” strongly disagreed and “5 “strongly agree. Indicators of the questionnaire scale variables are shown in Table 1.

**Table 1:** Items used to measure the constructs.

Constructs	Items	Contents	Source
Risk information characteristics (RIC)	RIC1	The hazard warning information I receive at the construction site is authentic and reliable	(51, 55)
	RIC2	I can be informed of dangerous situations or safety instructions at the construction site in a timely manner	
	RIC3	Safety notifications or warnings provide key information needed to deal with hazards without omission	
	RIC4	I trust and am willing to follow the safety information and instructions issued by the construction site authorities	
Information dissemination channels (IDC)	IDC1	I mainly rely on site announcements, safety meetings, and formal notifications from leaders/safety officers to obtain hazard information	(56–58)
	IDC2	I mainly obtain hazard information or related discussions through worker groups and mobile apps	
	IDC3	I mainly rely on oral notifications from team leaders, co-workers, or close co-workers to learn about hazard information and coping methods	
	IDC4	I mainly trust and refer to safety tips or training materials issued by professional departments such as fire departments and safety supervision	
Risk perception (RP)	RP1	I believe that the potential accidental consequences at the construction site are very serious	(7, 59, 62)
	RP2	I feel that the risk of accidents in my position or working environment is high	
	RP3	When I learn that a hazard is approaching, I believe immediate action must be taken	
	RP4	When I think about potential accidents, I feel worried and anxious	
Information self-efficacy (ISE)	ISE1	I am able to understand the various types of safety information and warning content I receive	(63, 65)
	ISE2	I am able to judge the authenticity and reliability of the safety information I obtain	
	ISE3	I know how to use safety information to protect my own safety	
	ISE4	Even if the information is complex or the situation is urgent, I am confident in processing the relevant information and responding	
Emergency information behavior (EIB)	EIB1	I will actively search for more information related to safety risks	(7, 67, 69–71)
	EIB2	I will share important safety information or warnings with my co-workers	
	EIB3	I will verify suspicious safety information by asking safety officers or checking official notifications	
	EIB4	I will act in accordance with formal safety instructions and procedural requirements	

### 4.2 Data collection

The questionnaire used in this study, distributed via WeChat and Wenjuanxing (Questionnaire Star) platforms, was designed based on the five constructs of the research model, comprising 25 relevant questions. We randomly selected 600 construction workers from different large-scale buildings construction projects in Hangzhou to participate. The study exclusively targeted high-rise residential/commercial buildings during structural erection stages. Participants were informed that clicking the “Submit Answer” button constituted their informed consent. Over the survey period (from March 3, 2025 to May 9, 2025), 600 questionnaires were distributed. After excluding 31 incomplete questionnaires, 569 valid responses were obtained. Descriptive statistical analyses were conducted on the collected 569 questionnaires, with the detailed results presented in Table 2.

**Table 2:** Survey statistics from questionnaires.

Variable	Items	Frequency	Percentage (%)
Gender	Male	490	86.1
	Female	79	13.9
Age	Under 25	16	2.8
	26–35	102	17.9
	36–45	140	24.6
	46–55	197	34.6
	Over 55	114	20.0
Work experience	Under 1 year	75	13.2
	1–3 years	145	25.5
	4–6 years	106	18.6
	7–10 years	119	20.9
	Over 10 years	124	21.8
Educational level	Primary school or below	130	22.8
	Junior high school	281	49.4
	High school/ Technical school	82	14.4
	Junior college	60	10.5
	Undergraduate and graduate degrees	16	2.8
Job category	Reinforcement Worker	98	17.2
	Carpenter	82	14.4
	Scaffolder	43	7.6
	Electrician	29	5.1
	Other	317	55.7

**Figure 3:**
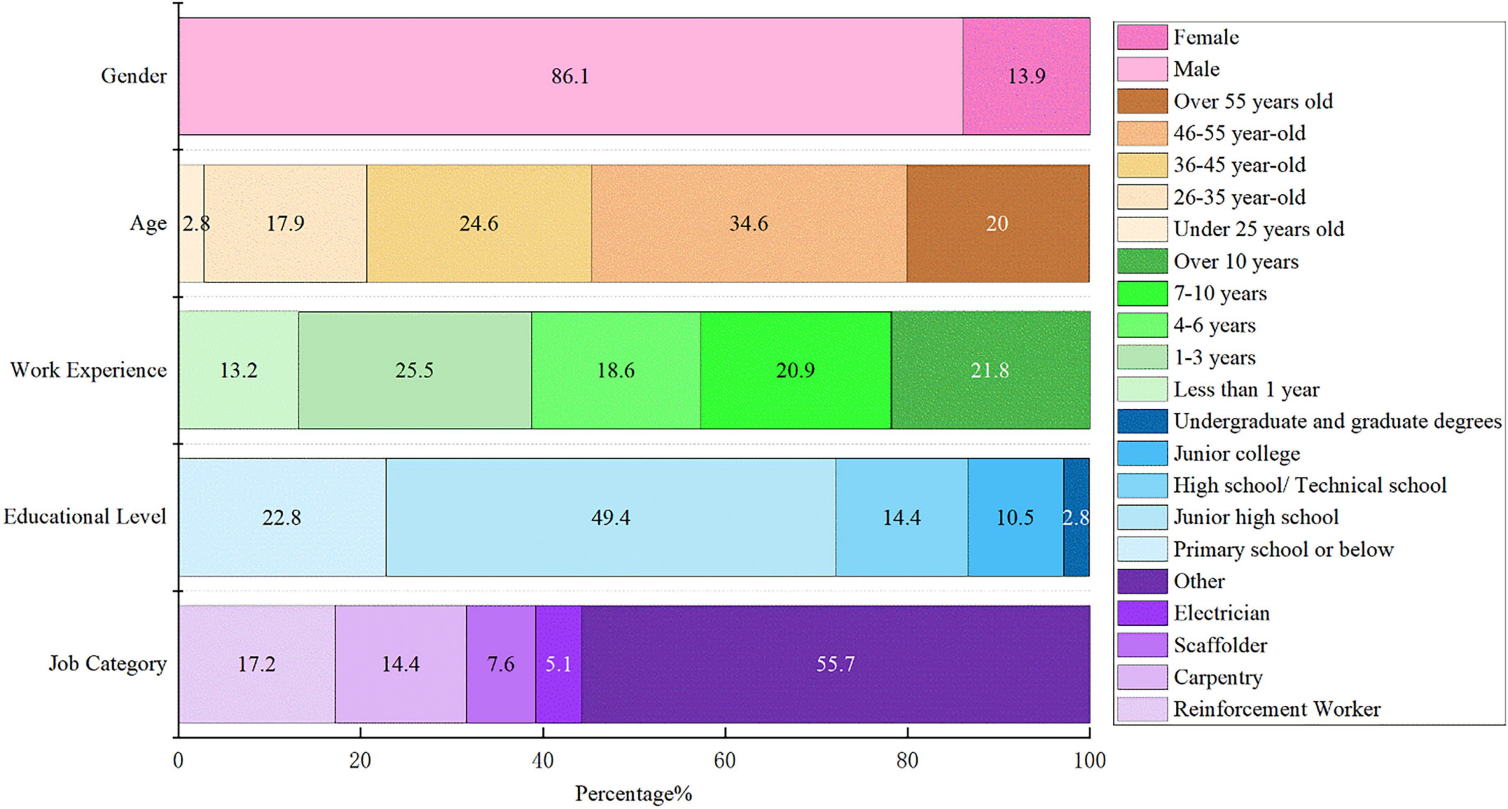
Participants’ demographic information.

Figure 3 visually reveals demographic information:

1. Gender ratio: 86.1% of respondents were male.
2. The age distribution survey shows that the majority of respondents are middle-aged, aged between 35 and 55.
3. Regarding educational attainment, the majority of respondents had completed post-secondary education or below, and a relatively small proportion held a bachelor’s degree or above.
4. A study of the respondents’ work experience in the construction industry showed that 39.5% had 4–10 years of experience and relatively few had more than 10 years.
5. The survey targeted construction workers on-site. And the survey on the types of work found that the proportion of other types of work besides steelworkers, carpenters, framers and electricians was as high as 55.7%. These findings coincide with the current situation of construction workers in Hangzhou, China, and to some extent increase the validity and reliability of the questionnaire data.

### 4.3 Method

**Figure 4:**
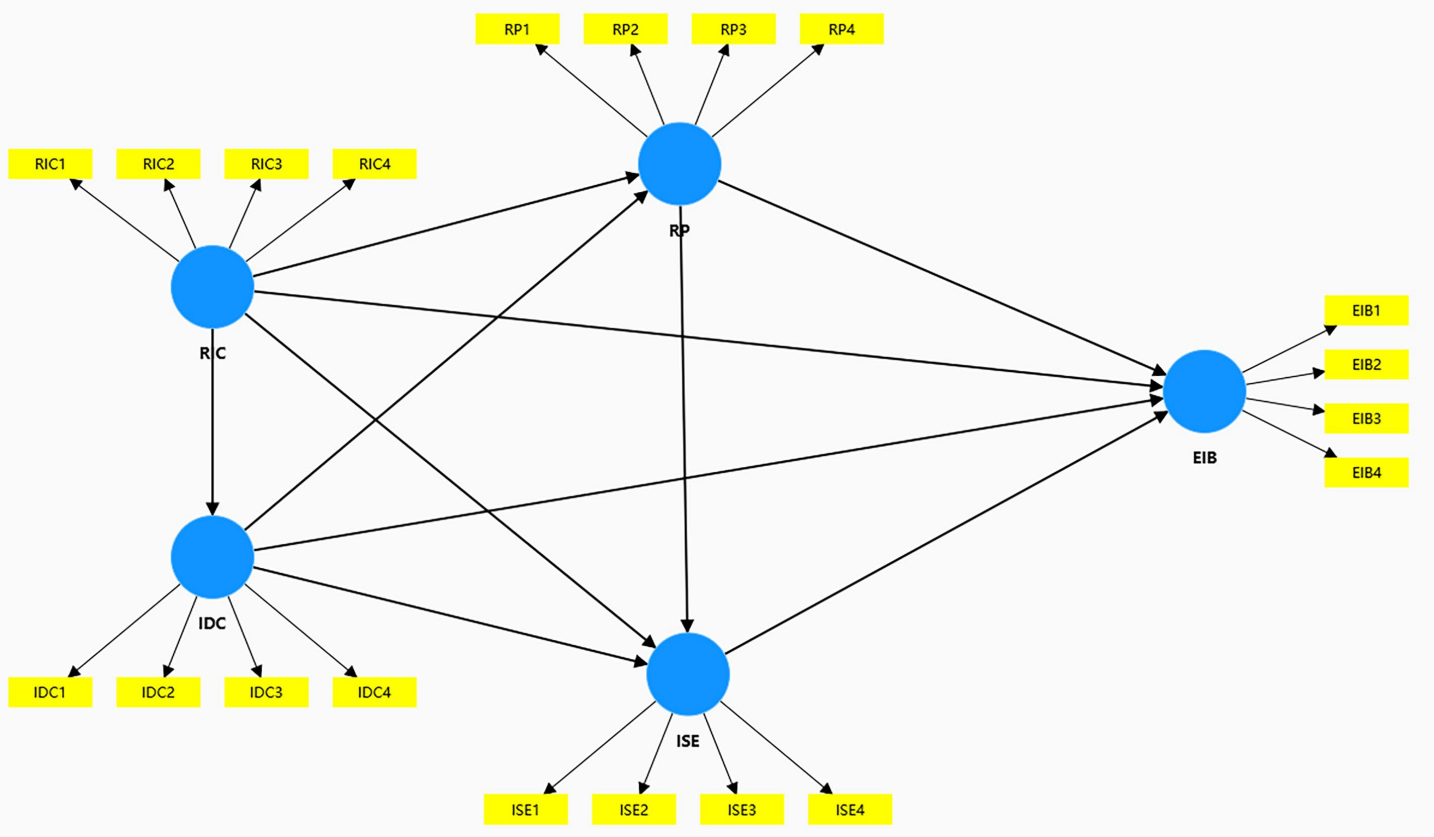
Partial least squares structural equation modeling.

Structural Equation Modeling (SEM) (76) was developed by Swedish statistician Karl G·Joreskog in the 1970s. Partial Least Squares SEM (PLS-SEM) (77) combines principal component analysis and ordinary least squares regression to predict key target variables. PLS-SEM handles small samples, non-normal data, and complex multi-construct models (78), widely used in management, MIS, and public administration (79, 80). The study’s methodological soundness is strongly supported by its sample size, which is fully adequate for the Partial Least Squares Structural Equation Modeling (PLS-SEM) analysis conducted. With 569 valid responses, the research operates on a dataset that is not only sufficient but robust. While PLS-SEM is recognized for its effectiveness even with smaller samples, this study’s large sample size far exceeds typical requirements, providing it with significant statistical power. This ensures that the findings are stable and that the relationships identified between variables are highly reliable. PLS-SEM testing involves measurement and structural model evaluation via composite reliability, Cronbach’s alpha, and latent variable AVE (81). The PLS-SEM model is shown in Figure 4.

### 4.4 Rationale for a reflective measurement model specification

A critical decision in measurement theory is the specification of constructs as either reflective or formative. While some constructs in our model, such as Information Dissemination Channels (IDC) and Risk Information Characteristics (RIC), could be conceptualized as formative, we have deliberately specified all constructs as reflective in this study. This decision is based on our theoretical focus on capturing the holistic subjective perceptions of workers.

We posit that the indicators for each construct are manifestations of a single, underlying latent variable. For instance, for Information Dissemination Channels (IDC), we measure a worker’s “overall perceived reliance on the information environment,” which is then reflected in their reported reliance on various specific channels. Similarly, for Risk Information Characteristics (RIC), we measure the “overall perceived quality of risk information,” which is reflected in its attributes like accuracy, timeliness, completeness, and credibility. This reflective approach assumes that the indicators for each construct should be correlated, as they are all influenced by the same underlying concept.

This theoretical stance is empirically supported by our data, as the subsequent measurement model evaluation demonstrates strong internal consistency for all constructs, which is a key characteristic of reflective models. While a formative approach represents a valid alternative for future research, the reflective specification aligns with the theoretical goals and perceptual focus of the present study.

## 5 Results

### 5.1 Measurement model evaluation

#### 5.1.1 Convergence validity

This study first tested whether items measuring the same construct had internal reliability. Four criteria were used to evaluate the results of reflective constructs: composite reliability (CR), average variance extracted (AVE), Cronbach’s alpha, and factor loadings. Composite reliability and Cronbach’s alpha values should be greater than 0.7, AVE values greater than 0.5, and factor loadings greater than 0.7. Table 3 shows the convergent validity results of indicators in our research model. All indicators’ composite reliability (CR) was greater than 0.9, AVE greater than 0.6, Cronbach’s alpha greater than 0.8, and factor loadings significantly greater than 0.7. In summary, these results indicate that our measurement model has satisfactory convergent validity, reflecting high internal consistency, reliability, and focus.

**Table 3:** The convergence validity of constructs.

Constructs	Items	Loading	Cronbach’s alpha	CR	AVE
Emergency information behavior	EIB1	0.884	0.890	0.924	0.751
	EIB2	0.874			
	EIB3	0.861			
	EIB4	0.848			
Information dissemination channel	IDC1	0.900	0.887	0.922	0.748
	IDC2	0.859			
	IDC3	0.854			
	IDC4	0.845			
Information self-efficacy	ISE1	0.884	0.853	0.901	0.695
	ISE2	0.836			
	ISE3	0.790			
	ISE4	0.822			
Risk information characterization	RIC1	0.876	0.859	0.905	0.703
	RIC2	0.820			
	RIC3	0.833			
	RIC4	0.825			
Risk perception	RP1	0.889	0.884	0.920	0.741
	RP2	0.860			
	RP3	0.844			
	RP4	0.850			

#### 5.1.2 Distinguishing validity

For measures of discriminant validity we used three methods-extracting the square root of the average variance, cross loadings and heteroskedasticity-monotone variance (HTMT) correlation ratios (82). Table 4 shows the square root of AVE for each structure greater than its correlation. Table 5 provides indicator loading values, which are higher than the cross-loading values of the items used to measure the other indicators. Table 6 gives the values of HTMT correlation ratios for each reflection configuration below the critical value (0.85). Thus, these results demonstrate the good discriminant validity of our measurement model.

**Table 4:** The correlations between constructs with reflective constructs.

Constructs	AVE	EIB	IDC	ISE	RIC	RP
EIB	0.751	0.867				
IDC	0.748	0.631	0.865			
ISE	0.695	0.592	0.606	0.834		
RIC	0.703	0.641	0.623	0.572	0.839	
RP	0.741	0.645	0.646	0.598	0.641	0.861

**Table 5:** The loadings and cross-loadings of reflective constructs.

	EIB	IDC	ISE	RIC	RP
EIB1	0.884	0.562	0.533	0.550	0.554
EIB2	0.874	0.548	0.505	0.546	0.548
EIB3	0.861	0.532	0.520	0.571	0.566
EIB4	0.848	0.546	0.492	0.553	0.568
IDC1	0.558	0.900	0.573	0.565	0.587
IDC2	0.524	0.859	0.477	0.493	0.515
IDC3	0.532	0.854	0.526	0.566	0.574
IDC4	0.566	0.845	0.512	0.526	0.551
ISE1	0.491	0.501	0.884	0.481	0.496
ISE2	0.488	0.484	0.836	0.483	0.478
ISE3	0.468	0.476	0.790	0.421	0.485
ISE4	0.522	0.552	0.822	0.517	0.530
RIC1	0.560	0.551	0.482	0.876	0.526
RIC2	0.498	0.481	0.456	0.820	0.496
RIC3	0.498	0.500	0.450	0.833	0.526
RIC4	0.584	0.552	0.525	0.825	0.595
RP1	0.558	0.559	0.524	0.555	0.889
RP2	0.569	0.583	0.533	0.564	0.860
RP3	0.556	0.516	0.489	0.547	0.844
RP4	0.538	0.563	0.511	0.542	0.850

**Table 6:** The heterotrait–monotrait (HTMT) ratio of correlations of reflective constructs.

Constructs	EIB	IDC	ISE	RIC	RP
EIB	/				
IDC	0.710				
ISE	0.678	0.692			
RIC	0.730	0.710	0.664		
RP	0.727	0.727	0.687	0.733	/

#### 5.1.3 Common methodological biases

Given that our data were collected from a single source via self-reported questionnaires, we took several procedural steps during the questionnaire design to minimize common methodological biases (CMB), such as ensuring anonymity and varying the item order. To statistically assess whether CMB was a significant concern in our final dataset, we employed a more robust and contemporary technique recommended for PLS-SEM: the full collinearity assessment.

This method involves calculating the Variance inflation factor (VIF) for all constructs in the model. If a substantial level of common method bias exists, it will manifest as high levels of collinearity among the constructs, leading to VIF values greater than the conservative threshold of 3.3 (83). We conducted this test by regressing each construct on all other constructs in the model.

As shown in Table 7, the results of the full collinearity assessment revealed that the VIF values for all constructs ranged from 1.768 to 2.905, with all values falling well below the 3.3 threshold. This provides strong evidence that common method bias is not a significant issue in our study, thereby enhancing our confidence in the validity of the reported structural relationships.

**Table 7:** Indicator collinearity assessment (VIF).

Constructs	VIF	Constructs	VIF	Constructs	VIF	Constructs	VIF	Constructs	VIF
EIB1	2.617	IDC1	2.905	ISE1	2.672	RIC1	2.447	RP1	2.700
EIB2	2.480	IDC2	2.428	ISE2	2.147	RIC2	1.918	RP2	2.257
EIB3	2.282	IDC3	2.270	ISE3	1.768	RIC3	2.049	RP3	2.145
EIB4	2.136	IDC4	2.168	ISE4	1.780	RIC4	1.792	RP4	2.149

### 5.2 Structural model evaluation

**Figure 5:**
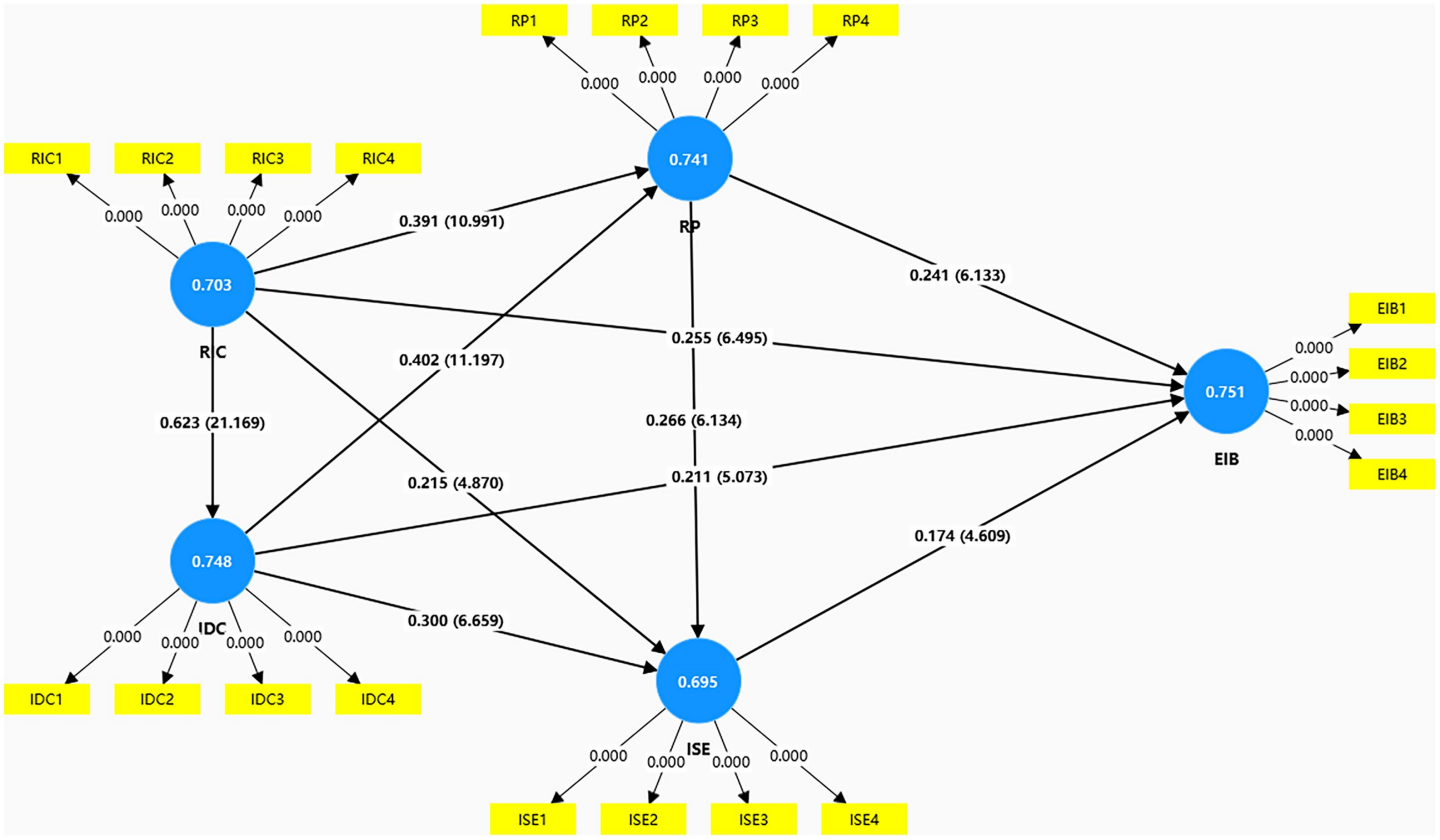
Structural equation model path coefficient diagram.

We used the bootstrap method (84, 85) for 5,000 samples to obtain the effectiveness of the SmartPLS 4.0 software based on structural modeling. As shown in Table 8 (path coefficients) and Table 9 (Goodness of Fit, GOF), they are used to test the hypotheses in the previous section. In Table 8, it is clear that all path coefficients are significant at the *p* < 0.001 level and the effect size of each path is above 0.02. This indicates that the structural model measures are statistically significant. For the structural model’s goodness of fit (Table 9), *R*^2^ assessed 0.482, 0.388, 0.464, and 0.510 for the emergency information behavior, information dissemination channel, information efficacy perception, and risk perception variables, respectively; this indicates that the model explained 48.2% of the variance in emergency information behavior, 38.8% of the variance in information dissemination channel, 46.4% of the variance in information efficacy perception, 51% of the risk perception variance. The model has moderate explanatory power in terms of willingness to engage in emergency information behavior. Meanwhile, *Q*^2^ values were also obtained in SmartPLS 4.0 by using a blindfold procedure and setting the *d* value to 7. The *Q*^2^ values of the four endogenous latent variables were all greater than zero (the minimum value was 0.287), indicating that these endogenous variables gained predictive relevance. According to Wetzels et al. (85) the three critical values of the GOF are, in order, 0.10, 0.25, 0.36, when the value of GOF is between 0.10 and 0.25, the model fit is weak; when the value of GOF is between 0.25 and 0.36, the model fit is moderate; when the value of GOF is large 0.36, the model fit is good. The calculated GOF value of the model in this study is 0.591, which is greater than 0.36, so the model in this study has a good fit. In summary, the theoretical paths (H1 to H10) that we assumed in Figure 2 are acceptable. The results show that risk information characteristics, information dissemination channels, risk perception, and information efficacy perception constructs have a positive effect on emergency information behavior constructs, and risk perception has a positive effect on information efficacy perception, contrary to the initial negative hypothesis H7, risk perception and information efficacy perception constructs have a significant mediating effect on emergency information behavior constructs. The path diagram of the structural equation model is shown in Figure 5.

**Table 8:** Results of path coefficients and hypothesis testing.

Hypotheses	Path coef.	St. error	*T* values	95% CI		*f*^2^	Result
				2.5%	97.5%		
H1: RIC → RP	0.391	0.036	10.991	0.320	0.457	0.191	Found
H2: RIC → ISE	0.215	0.044	4.870	0.128	0.300	0.044	Be tenable
H3: IDC → RP	0.402	0.036	11.197	0.332	0.474	0.202	Found
H4: IDC → ISE	0.300	0.045	6.659	0.213	0.388	0.085	Found
H5: RP → EIB	0.241	0.039	6.133	0.163	0.320	0.060	Found
H6: ISE → EIB	0.174	0.038	4.609	0.099	0.246	0.036	Found
H7: RP → ISE	0.266	0.043	6.134	0.180	0.348	0.065	Not established
H8: RIC → EIB	0.255	0.039	6.495	0.176	0.330	0.072	Found
H9: IDC → EIB	0.211	0.042	5.073	0.131	0.294	0.047	Found
H10: RIC → IDC	0.623	0.029	21.169	0.560	0.675	0.634	Found

**Table 9:** The validity of the structural model.

Construct	EIB	IDC	ISE	RP
*R*^(2)^ (coefficient of determination)	0.555	0.388	0.464	0.510
*Q*^(2)^ (predict relevance)	0.412	0.287	0.318	0.373
GOF (goodness of fit)	0.591			

**Figure 6:**
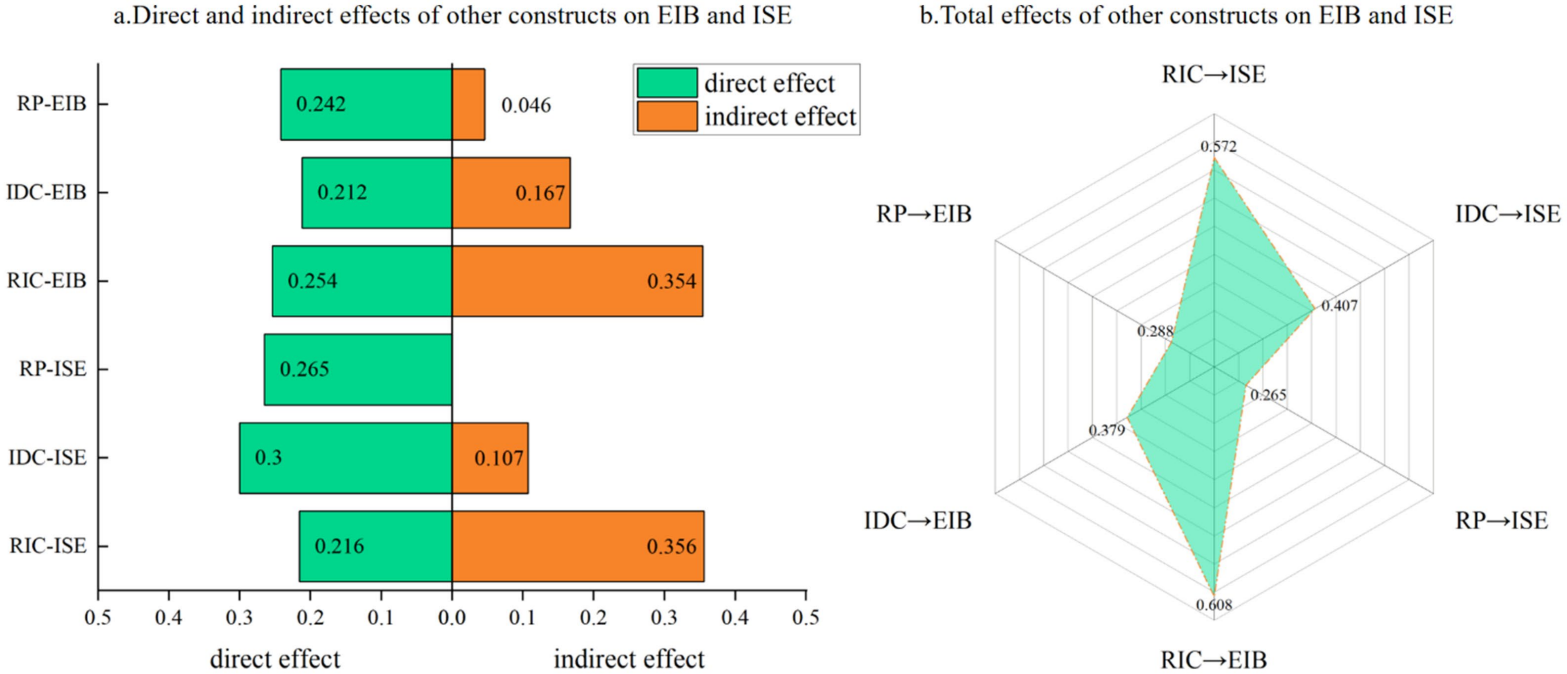
The effects of RIC, IDC, and RP on ISE and EIB. **(a)** Direct and indirect effects. **(b)** Total effects.

Figure 6 compares the direct, indirect, and total effects of the other constructs in our model on the sense of information efficacy and contingent information behaviors. The total effect of IDC on ISE (0.407) is higher than that on EIB (0.379). The total effect of RIC on EIB (0.608) is higher than that on ISE (0.572). The total effect of RP on EIB (0.288) was higher than the total effect on ISE (0.265). RP, IDC, RIC had a direct effect on both ISE and EIB, IDC and RIC had an indirect effect on both ISE and EIB, while RP had an indirect effect only on EIB.

### 5.3 Multi-group analysis

#### 5.3.1 Cross-work experience comparison

This study explores the impact of working age on a group of construction workers through a multi-group comparative analysis approach. In order to accurately capture the effect of the age variable, the sample was divided into five age groups: less than 1 year of service (G1), 1–3 years of service (G2), 4–6 years of service (G3), 7–10 years of service (G4), and more than 10 years of service (G5). Based on the critical characteristics of industry experience accumulation, G4 and G5 were combined to form the “more than 7 years of service” group (GC) as the control group. This is due to the fact that this group has mature industry stability: not only have they gone through the complete cycle of skill formation (usually 7 years is the critical threshold), but they also represent the industry benchmark in terms of enforcing safety regulations, handling complex working conditions, and occupational identity, etc. The GC group serves as a reference point to identify the differences in the characteristics of the other age groups (G1–G3) in comparison to the senior workers. The age group maps the career development trajectory: G1 (adaptation period) has skills and norms recognition gaps; G2–G3 (growth period) shows skills enhancement and role differentiation; and the GC group (stabilization period) embodies experience consolidation, technical authority status, and risk avoidance tendency.

Other path coefficients and differences were calculated but not shown; Table 10 presents an excerpt. The study found significant differences in information processing and behavioral responses among construction workers of different experience levels. In the four groups, except for “RIC → ISE,” coefficients on most paths were significantly different. The GC group showed stronger positive effects of ISE on EIB (0.174, *p* = 0.000), RP on EIB (0.226, *p* = 0.000), and RP on ISE (0.382, *p* = 0.005). That is, the impact of IDC and RP on emergency information behavior is more prominent among senior workers. However, the relationship between RIC and EIB (0.328, *p* = 0.006) and IDC and ISE (0.391, *p* = 0.000) was stronger in the G2 group, indicating that workers with some site experience are more sensitive to risk information and better at utilizing information dissemination channels.

**Table 10:** Multi-group analysis results by work experience.

Path	Coef. GC	Coef. G1	Coef.	Coef.	Diff’0.1	Diff’0.2	Diff’0.3
IDC → EIB	0.229	0.065	0.196	0.355	0.164	0.033	−0.126
IDC → ISE	0.299	0.347	0.391	0.178	−0.048	−0.092	0.121
ISE → EIB	0.174	0.103	0.084	0.124	0.071	0.090	0.050
RIC → EIB	0.230	0.322	0.328	0.142	−0.092	−0.098	0.088
RIC → ISE	0.080	0.390	0.343	0.223	−0.31	−0.263	−0.143
RP → EIB	0.226	0.223	0.218	0.199	0.003	0.008	0.027
RP → ISE	0.382	0.014	0.070	0.311	0.368	0.312	0.071

#### 5.3.2 Cross-education level comparison

This study divided construction workers into four groups by education level: primary school or below (E1), junior high school (control group, 62.7%), high school/technical school (E3), and higher education (E4, including college and above). Based on the distribution characteristics of human capital in the construction industry, the junior high school group (E2) was set as the control group. The E2 group serves as a reference baseline to identify differential characteristics of other education groups (E1, E3, and E4) relative to the main workforce. The E1 group (weak cognition period) has gaps in safety regulation understanding and technological upgrading barriers; the E3 group (skill strengthening period) shows quality control expertise and construction coordination potential; the E4 group (innovation leadership period) reflects technological integration capabilities and management authority; the E2 group (reference baseline) represents standard industry operation modes and stability needs.

As shown in Table 11. In the four groups, the coefficients on most pathways were significant and somewhat different, except for “IDC → EIB,” where the positive effects of ISE on EIB (0.218 *p* = 0.000), RIC on EIB (0.306, *p* = 0.000), and RP on EIB (0.232, *p* = 0.005) were much stronger in the E2 group have stronger positive effects. That is, the effects of ISE and RP on construction workers’ emergency information behavior are more prominent among workers with long years of service. However, the relationship between IDC on EIB (0.444, *p* = 0.006), and IDC and ISE (0.533, *p* = 0.000) was found to be stronger in the E4 group, suggesting that highly educated workers are the most sensitive to the information dissemination channels and are good at utilizing them, which in turn influences emergency information behavior.

**Table 11:** Multi-group analysis results by education level.

Path	Coef. E2	Coef. E1	Coef.	Coef. E4	Diff’0.1	Diff’0.2	Diff’0.3
IDC → EIB	0.155	0.131	0.281	0.444	−0.024	−0.15	−0.313
IDC → ISE	0.288	0.289	0.114	0.533	0.001	0.175	−0.244
ISE → EIB	0.218	0.164	0.118	0.216	−0.054	0.046	−0.052
RIC → EIB	0.306	0.262	0.272	0.159	−0.044	−0.01	0.103
RIC → ISE	0.138	0.242	0.331	0.137	0.104	−0.089	0.105
RP → EIB	0.232	0.226	0.218	0.163	0.034	0.008	0.103
RP → ISE	0.376	0.26	0.289	0.087	−0.116	−0.029	0.173

## 6 Discussion

This study’s findings elucidate the complex mechanisms governing construction workers’ emergency information behavior, with the structural model accounting for a substantial 55.5% of its variance (*R*^2^ = 0.555). A contextualized interpretation of the path coefficients reveals a nuanced interplay of informational inputs and psychological mediators that drive behavioral responses within this high-risk occupational setting.

Our analysis establishes Risk Information Characteristics (RIC) and Information Dissemination Channels (IDC) as foundational antecedents to workers’ cognitive states. Notably, these two exogenous constructs exert a powerful and statistically equivalent influence on Risk Perception (RP) (RIC → RP, *β* = 0.391; IDC → RP, *β* = 0.402). This parity suggests a synergistic relationship: the efficacy of high-quality information is contingent upon its delivery through robust, multi-faceted channels, and vice versa. On a dynamic worksite, neither a well-crafted message in isolation nor a multi-channel blast of ambiguous information is sufficient to meaningfully elevate situational awareness.

A more differentiated pattern emerges in the formation of Information Self-Efficacy (ISE). Here, the influence of IDC (*β* = 0.300) is markedly stronger than that of RIC (*β* = 0.215). This finding suggests that for this specific demographic, the modality and accessibility of information channels are more critical to fostering confidence than the intrinsic attributes of the information itself. Interactive and trusted channels, such as a supervisor-moderated mobile messaging group, appear to provide a sense of agency and verification that directly enhances a worker’s perceived capacity to manage an emergency, underscoring the importance of dialogic rather than merely didactic communication systems.

Perhaps the most theoretically significant finding pertains to the dual pathways originating from Risk Perception (RP). Consistent with established theory, RP serves as a direct and robust impetus for Emergency Information Behavior (EIB, *β* = 0.241). However, contradicting the cognitive overload hypothesis, RP also functions as a powerful positive antecedent to ISE (*β* = 0.266). This counterintuitive positive relationship suggests that for this resilient occupational group, a heightened appraisal of risk acts as a motivational catalyst rather than a cognitive inhibitor. The perception of a tangible threat appears to trigger a proactive, problem-solving orientation, thereby enhancing an individual’s belief in their own capability. This “threat-to-capability” pathway represents a crucial psychological mechanism for resilience in high-risk environments.

While these core pathways are generalizable, our multi-group analysis reveals that their efficacy exhibits significant heterogeneity across worker subgroups. We interpret these differences not as isolated findings, but within the frameworks of Career Stage Theory and Information Literacy Theory, which allows us to map an “evolutionary trajectory” of workers’ behavioral drivers. For instance, less experienced workers (1–3 years) demonstrated the highest sensitivity to external cues, namely the quality of Risk Information (RIC) and Dissemination Channels (IDC). This aligns with early career stages, where individuals rely more heavily on explicit, authoritative information to guide their actions. Conversely, the behavior of highly experienced senior workers (≥7 years) was more strongly predicted by their internal assessments, namely Risk Perception (RP) and Information Self-Efficacy (ISE). This suggests that as experience accumulates, external information is gradually internalized into an intuitive risk-assessment capability. Similarly, differences in education level reflect varying degrees of information literacy, with more highly educated workers proving more adept at leveraging diverse channels (IDC) to empower their behavior.

In summary, our model offers a theoretically nuanced and empirically grounded guide for enhancing safety on construction sites. The findings advocate for a holistic and stratified strategy. To cultivate a resilient and proactive workforce, management must not only disseminate high-fidelity information (RIC) through diverse and interactive channels (IDC) but also leverage the motivational power of risk perception (RP). By consistently pairing risk alerts with clear, actionable solutions, and by tailoring the communication approach to the experience and education of the workforce, organizations can harness workers’ intrinsic response mechanisms, transforming perceived threats from potential stressors into catalysts for empowered, safe behavior.

## 7 Conclusion

This study explored the comprehensive mechanism of associations between risk information characteristics, dissemination channels, risk perception, information self-efficacy, and construction workers’ emergency information behavior. The results show that risk information characteristics (RIC)—encompassing accuracy, timeliness, completeness, and credibility—are significantly and positively associated with workers’ risk perception (*β* = 0.391) and information self-efficacy (*β* = 0.215), and are also a direct positive predictor of emergency information behavior (*β* = 0.255). This indicates that high-quality risk information is a core factor related to stimulating workers’ risk awareness and information actions. Concurrently, diversified dissemination channels (IDC) were also found to be significantly and positively associated with risk perception (*β* = 0.402) and information self-efficacy (*β* = 0.300), and had a direct positive relationship with emergency behavior (*β* = 0.211), highlighting the critical value of social and interpersonal networks in expanding information coverage.

The model suggests that risk perception is not only a direct predictor of emergency information behavior (*β* = 0.241) but is also indirectly linked to it through its positive association with information self-efficacy (*β* = 0.266), while information self-efficacy itself is a significant predictor of behavior (*β* = 0.174). This particular finding offers a significant insight, as it contradicts our initial hypothesis (H7) grounded in the theory of cognitive overload. The strong positive relationship found in our data suggests that for construction workers, the motivational aspect of risk perception is more prominent than the potential for cognitive impairment. A heightened awareness of danger appears to be related to a proactive mindset, strengthening workers’ belief in their own ability to manage and act upon emergency information. This finding underscores the psychological resilience of this occupational group.

Furthermore, the group heterogeneity analysis provided a more nuanced view of these mechanisms, revealing a clear evolutionary pattern of behavioral drivers consistent with career stage and information literacy theories. The findings indicate a distinct shift in reliance from external informational cues, such as information quality and channels among novice workers, to a dependence on internalized, experience-based factors like risk perception and self-efficacy among their senior counterparts. This progression underscores the necessity of moving beyond one-size-fits-all communication strategies.

The study also validated the strong positive association between RIC and IDC (*β* = 0.623), revealing that high-quality information has a higher likelihood of multi-channel dissemination. These findings lead to several actionable practical implications for enhancing emergency management in the construction industry. Our results advocate for a move beyond generic safety protocols toward a more precise and psychologically-informed approach. The foundational importance of RIC calls for the establishment of rigorous, multi-tiered information vetting systems to ensure all safety alerts are accurate, complete, and credible. Information systems should be designed to deliver highly contextualized, formatted alerts that specify the nature of the risk and the required action, thereby transforming abstract warnings into empowering, actionable intelligence.

Concurrently, the critical role of IDC necessitates an integrated and interactive communication strategy. We recommend a “1 + 1 + N” model: one primary authoritative channel, one core instant-messaging group, and numerous team leaders acting as trusted information nodes. This structure leverages both formal authority and informal, interpersonal networks. Finally, the heterogeneity revealed by our multi-group analysis calls for stratified and targeted interventions. For example, simulation-based drills are most effective for apprentices who rely on external guidance, while peer-led risk debriefing sessions may be more impactful for seasoned veterans driven by internalized experience. By implementing these data-driven strategies, firms can translate research insights into a more resilient and proactive safety culture. Future research should explore these behavioral differences across job types and integrate macro variables like organizational culture, with a rigorous cost–benefit analysis representing a logical next step.

## 8 Limitations and future research

This study, while offering valuable insights, is subject to several limitations that provide clear directions for future inquiry. First, the geographic concentration of our sample to construction workers in Hangzhou necessitates caution when generalizing findings to a national or multi-industry context. Similarly, the study’s scope was limited regarding the diversity of job types and a thorough examination of gender-based differences.

A primary methodological limitation, however, is the study’s cross-sectional research design. The reliance on a single point-in-time, self-reported survey inherently restricts our ability to capture the dynamic evolution of behavior and, more importantly, precludes the establishment of temporal precedence necessary for inferring causality. Consequently, while our path analysis supports the proposed relationships, terms such as “effect” or “impact” used throughout this paper should be interpreted as statistically significant associations rather than confirmed causal pathways. This constraint is particularly salient for our mediation analyses. Although our findings are consistent with a mediational framework, the cross-sectional data only captures a static snapshot of these relationships. We cannot empirically confirm that changes in informational factors precede changes in the mediators, which in turn precede behavioral responses. Therefore, the “indirect effects” we identified represent statistically supported correlational chains, not validated causal processes.

From a modeling perspective, our framework did not fully integrate organizational context variables, such as safety culture or specific training programs. We also acknowledge that our deliberate choice to specify all constructs as reflective, while aligned with our focus on holistic perceptions, is a key limitation. A valuable avenue for future research would be to employ a formative measurement specification for constructs like Information Dissemination Channels (IDC) to offer a complementary view of how their distinct elements form a whole. Moreover, the internal mechanism driving the counter-intuitive positive link between risk perception and information self-efficacy remains to be fully explicated. The study’s scope was also intentionally focused on behavioral dynamics, leaving formal economic analysis for subsequent research.

Accordingly, future studies should prioritize sample diversification and, crucially, the use of research designs that can address the issue of causality. This includes employing longitudinal tracking to test the causal ordering of our model and using experimental designs to isolate the causal impact of specific informational interventions. A deeper analysis of the psychological pathway linking risk perception to self-efficacy across various emergency scenarios is also warranted. On a practical level, we recommend enhancing the integration of smart technologies on construction sites and developing tailored training programs. Such evidence-based strategies, though requiring upfront investment, promise to reduce the severe human and financial costs of emergencies, underscoring the need for economic feasibility studies as a vital next step.

## Data Availability

The raw data supporting the conclusions of this article will be made available by the authors, without undue reservation.
